# Role of pharmacometrics and systems pharmacology in facilitating efficient dose optimization in oncology

**DOI:** 10.1002/psp4.13066

**Published:** 2023-11-03

**Authors:** Priya Jayachandran, Rajat Desikan, Sriram Krishnaswami, Stefanie Hennig

**Affiliations:** ^1^ Regeneron Pharmaceuticals, Inc. Tarrytown New York USA; ^2^ Clinical Pharmacology Modeling & Simulation GlaxoSmithKline (GSK) Stevenage Hertfordshire UK; ^3^ Oncology Research and Development Pfizer Groton Connecticut USA; ^4^ Certara, Inc. Melbourne Victoria Australia; ^5^ School of Clinical Sciences, Faculty of Health Queensland University of Technology Brisbane Queensland Australia

As one of three journals within the ASCPT family of publications, *Clinical Pharmacology and Therapeutics: Pharmacometrics and Systems Pharmacology* (*CPT:PSP*) is committed to publishing top‐quality work that introduces and applies innovative quantitative methods to support drug discovery and development. Whereas *PSP* has long been a key component in oncology discovery, development, regulatory approval, and clinical practice, this themed issue focusing on dose optimization in oncology was envisioned because of the renewed interest in oncology dose selection and optimization, triggered by the Project Optimus initiative in 2021 by the US Food and Drug Administration (FDA).^1^ Our call for papers invited original research, tutorials, reviews, and perspectives, all with the intent of providing an early pulse of how *PSP* approaches facilitate dose optimization in oncology, and we are pleased to note the positive interest and response from the oncology community on this hot topic.

The articles in the themed issue can be broadly classified into three categories: (1) continuation of core/traditional quantitative clinical pharmacology applications (e.g., population pharmacokinetics [PK] and PK/pharmacodynamics [PD]) to characterize dose/exposure‐response (ER) relationships enabling dose optimization, (2) newer quantitative modeling and simulation methodologies (e.g., machine learning [ML], quantitative systems pharmacology [QSP], and model‐based meta‐analyses [MBMA] among others) for informing dose and biomarker selection, and (3) model‐informed drug development (MIDD) strategies for rational clinical trial design.

Tosca et al.^2^ illustrate a translational model‐based approach integrating PK and tumor growth inhibition (TGI) data in mice to extrapolate a range of minimum effective concentrations for MEN1611, a compound in clinical development in combination with trastuzumab for patients with breast cancer. Using the PK/PD‐TGI model built to characterize the PK/PD relationship for the combination therapy, the minimum target exposure for tumor eradication was proposed and confirmed in an ongoing phase Ib study illustrating an effective use of *translational* PK/PD models to predict target exposures from preclinical data. Hodson et al.^3^ extend the application of the new dosing paradigm to triple therapy, using a mathematical model to fit preclinical data to identify optimal doses for radiotherapy in combination with immune checkpoint inhibitors and inhibitors of the DNA Damage Response Pathway. The model, which incorporates measures of cellular dynamics to characterize antigen presenting cell activation by radiotherapy and the effect of combination therapy on immune response to describe the impact of tritherapy on T cells and target tumor cells, is used to simulate instances of optimal tritherapy efficacy.

Moving from translational to clinical development, Guo et al.^4^ provide an example of how integration of early clinical PK, biomarker, safety, and tolerability data from a dose escalation and expansion study using semimechanistic population PK/PD modeling analyses can be used to guide selection of a single recommended phase II dose that optimizes the benefit–risk balance using the totality of available data. Akin to the holistic approach used by Guo et al.,^4^ Xu et al.^5^ demonstrate an effective utilization of modeling analyses to recommend dose regimens for phase II/III studies. In the absence of a well‐established biomarker reflecting sabatolimab efficacy to inform dose selection, Xu et al.^5^ develop a modeling and simulation strategy for PK and target occupancy to describe the ER relationship for safety and efficacy for sabatolimab monotherapy and combination therapy. Gong et al.^6^ used a traditional population PK and ER analysis approach to identify a dosing regimen with a favorable benefit–risk profile.

Morcos et al.^7^ illustrate how fundamental principles of the new dosing paradigm, namely ER and exposure‐safety analyses, may be applied to study a combination therapy of an already approved monotherapy, copanlisib, which used maximum tolerated dose (MTD) as the basis for dose selection rather than dose‐finding studies, with rituximab. Through their analysis, the authors find that a lower copanlisib dose results in lower efficacy, but without gains in safety and tolerability. Connarn et al.^8^ present a distinctive example of an integrated efficacy and safety ER analysis for a novel chimeric antigen receptor (CAR) T cell therapy for patients with relapsed and refractory multiple myeloma. Adoptive cell therapies have unique challenges; they are delivered once making the determination of optimal exposure a high priority, and cellular proliferation following drug administration can complicate the understanding of the dose‐exposure relationship, which is also highlighted by Mc Laughlin et al.^9^ Connarn et al.^8^ used ER models for efficacy end points (overall response rate [ORR] and complete response rate) and safety events (cytokine release syndrome [CRS]) to simulate dose–response relationships and demonstrate a positive benefit–risk assessment.

Yates et al.^10^ return to the three pillars of clinical pharmacology to argue their importance for dose optimization, specifically that PKs, target binding, pharmacology, and a fourth pillar, disease time course, are essential to understand the relationship between dose and efficacy. They describe how PK/PD relationships (with variability) define the shape of the dose–response relationship and propose using the modeling analysis, rather than a range of predefined functional forms, to generate hypotheses, explore trial designs, and drive dose optimization beginning in the early phases of oncology drug development.

Marolleau et al.^11^ showed how pharmacometric application to real‐world patients' data can help not to “kill a fly with a sledgehammer.” Utilization of their routine therapeutic drug monitoring data in a modeling and simulation study showed that the dosing interval for atezolizumab could be extended greatly while still maintaining exposures above the target threshold.^11^

Transitioning to newer or non‐traditional approaches, we note the work by Gevertz and Kareva^12^ who introduce a new algorithm to predict drug synergy—Multi‐Objective Optimization of Combination Synergy – Dose Selection (MOOCS‐DS) – which decouples the synergies of potency and efficacy and identifies Pareto optimal solutions in a multi‐objective synergy space. Utilizing preclinical data, they demonstrate the potential of their approach to guide the dose and schedule selection of synergistic combinations of the PD‐1 checkpoint inhibitor pembrolizumab and the anti‐angiogenic drug bevacizumab. Biomarker identification and validation is also key to designing effective clinical trial end points for combination therapies. Ojara et al.^13^ developed a Weibull time‐to‐event model to predict overall survival (OS) in patients receiving paclitaxel/platinum combination chemotherapy and decoded that a combination of two biomarkers – C‐reactive protein in blood, and tumor size at week 8 relative to baseline – most significantly affected OS. Just as important, they deduced that a range of biomarkers were either not significantly associated with OS or were significant only in univariate analyses in a subset of patients, thus enabling early individual prognostic predictions and treatment decisions. In another study, Weddel^14^ used a QSP model that mechanistically captured clinical cytokine dynamics following dosing with CD3‐based bispecifics in patients with solid tumors. The model predicted cytokine biomarker dynamics that could be indicative of CRS, a common clinical adverse effect upon CD3‐bispecific dosing, and therefore supports the selection of a safe dosing regimen for CD3‐bispecifics that also exhibits antitumor efficacy.

An issue with optimizing oncology drug therapies is that dose/exposure‐ efficacy responses are often derived separately for oncology end points, such as progression‐free survival (PFS) and OS. Liu et al.^15^ deploy a multistate pharmacometric modeling and simulation framework, developed to describe all end points including PFS and OS, and as a function of patient covariates including demographics, premedication, PK characteristics, and disease factors. Utilizing a clinical dataset of 80 patients with non‐small cell lung cancer (NSCLC) receiving two doses of bintrafusp alfa, the model decoupled confounding covariates and suggests that the higher dose led to better OS at the cohort level.

Utilizing historical data to benchmark the clinical efficacy of standard‐of‐care (SOC) therapies is critical for go/no‐go criteria in early phase oncology trials, where the efficacy of monotherapies can be compared against SOC, or combinations of novel drugs and SOC mandated by ethical/regulatory criteria require the decoupling of novel drug efficacy from that of SOC. Turner et al.^16^ utilize MBMA to analyze 15 published studies with PD‐1 inhibitors pembrolizumab and nivolumab, which represent the SOC in metastatic NSCLC. The advantage with MBMA lies in its ability to normalize differences across multiple studies/trials. The analysis by Turner et al.^16^ demonstrates associations between ORR and OS as well as the effect of covariates including squamous histology, PD‐1 tumor expression status, and type of therapy (mono vs. combination) in patients with NSCLC. This quantitative benchmarking methodology can be applied widely in oncology and facilitates accurate early‐phase decisions that are expected to reduce the failure rate of late‐phase trials. In another study, Hughes et al.^17^ introduced a hybrid PK/PD‐ ML framework to enable more precise individual‐level predictions of chemotherapy‐induced neutropenia. The ML models were trained on real‐world data from electronic health records augmented by synthetic data generated with the PK/PD model. The PK/PD‐enrichment of ML training datasets improved prediction of grades 3–4 neutropenia and serve as an example application of predictive modeling and simulations at the interface of clinical pharmacology and artificial intelligence and ML.

A few articles in this special issue have looked at deploying MIDD strategies for rational clinical trial design. The perspective by Shord et al.,^18^ co‐authored by several colleagues from the FDA, encourages new approaches to be embraced in oncology drug development and all available clinical and nonclinical data to be considered in real time, moving away from the MTD‐based paradigm. In their perspective, the authors suggest that particularly innovative trial designs and analytical iterative processes should play a greater role, as well as a delineated plan to robustly establish dose/ER relationships and well‐documented limitations when using a single dosage design. They also highlight that a “one‐size‐fits‐all” approach does not apply to every development program, and it certainly does not apply to all patients with cancer, therefore it is imperative that optimized dosages are identified for all patients before drug approval. They encourage early engagement with the FDA and highlight a key goal of Project Optimus, to plan for and discuss dose‐finding and optimization with the FDA early in the process.

One design approach was illustrated by Hooijmaijers et al.^19^ in their tutorial on stepwise development of an adaptive modeling and simulation workflow that can provide simulation‐driven insights, adapted dosing based on efficacy and/or safety end points or biomarkers. The availability of such simulation workflows, based on models developed and then updated throughout the MIDD phases, is becoming standard practice to quantitatively propose dosing regimens to guide discussion on dose optimization, not only in oncology drug development.

Shord et al.^18^ also suggested that strategically planned expansion cohorts, and randomized parallel dosage comparisons can yield additional clinical data to increase confidence, as well as early engagement with the regulatory agency to use a holistic approach. Norris^20^ has provided a statistical assessment of the consequences of the proposals for randomized dose‐finding trials by Project Optimus. Norris shows that even when designed and conducted under ideal circumstances, reasonably sized trials of the kind advocated by FDA's Oncology Center of Excellence (OCE) may need to enroll many hundreds of participants.^20^ Norris proposed a model of individual‐patient efficacy‐toxicity trade‐off, which highlights that there is a crucial difference between individual optimal dosing and population optimal dosing, which is a fundamental concept commonly disregarded during drug development. This original research article illustrates a formal model to help designers of oncology clinical trials to think more concretely and realistically about interindividual heterogeneity in dose‐efficacy trade‐offs.

The last two cases show how newer oncology treatment may not follow the traditional ER relationship. Mc Laughlin et al.^9^ reviewed autologous CAR‐T cell therapy development and how using MIDD approaches to enable study and program design choices when dose‐exposure relationships do not seem to exist for any of the currently approved CAR‐T cell products and consequently exemplifies a need for alternative approaches besides dose titration to optimize exposure. The review illustrates how considering all available clinical and especially early nonclinical data needs to be considered during the development and discovery process and how an MIDD program can help to quantitatively evaluate the impact on CAR‐T cell expansion, persistence, efficacy, and safety, and allow for more efficient candidate comparison to accelerate the development process. Sánchez et al.^21^ showed that similar difficulties to detect a clear dose–response relationship apply when developing bispecific antibodies. In their example, the bispecific antibody is exerting 4‐1BB‐associated T‐cell activation only while simultaneously bound to the FAP receptor. Using in vitro data together with mathematical modeling supported dose finding of clinical doses with an expected maximum of trimeric complex formation.

In conclusion, the FDA draft guidance on dose optimization was issued recently in January 2023, encouraging prospective randomized dose response trials and other approaches, and noting the continued importance of PSP to support dose selection. We are proud to publish such a rich themed issue promptly after issuance of the guidance. Although these are early days, the wide variety of applications in this themed issue underscore the potential for PSP to be a critical component of dose optimization efforts. We anticipate that innovative approaches will continue to be implemented in the next 2–3 years, and PSP and MIDD will further evolve to support efficient dose optimization, optimal benefit/risk and improved quality of life for patients. We are grateful to the reviewers, whose thoughtful and timely comments on the articles in this issue were invaluable. We welcome all readers to submit commentary on this editorial and/or on any of the articles of this themed issue and *CPT:PSP* welcomes further publications on this topic.

## FUNDING INFORMATION

No funding was received for this work.

## CONFLICT OF INTEREST STATEMENT

The authors declared no competing interests for this work.
